# Recent Dermatological Literature upon “New Formations.”

**Published:** 1879-06

**Authors:** Edward Wigglesworth

**Affiliations:** Boston, Mass., Dermatologist to the Boston City Hospital and Instructor in Syphilis at Harvard Medical School


ATLANTA

Medical and Surgical Journal.

Vol. XVII.] JUNE—1879. [No. 3

### Original Communications.

### RECENT DERMATOLOGICAL LITERATURE UPON "NEW FORMATIONS."

By EDWARD WIGGLESWORTH, M.D., Boston, Mass.,
Dermatologist to the Boston City Hospital and Instructor in Syphilis at Harvard Medical School.

*Treatment of Nævi by Multiple Scarifications.*—Following Balmanno Squire, Vidal has removed from the face and body various abnormal growths by means of scarification; large nævi diminished in size and small ones disappeared under this treatment from obliteration of vessels and cicatricial formation. After the fourth scarification, and at times earlier, the part operated on appears of a rosy instead of a purplish-red color, as before. Next small inlets form in the nævus which soon assume the color of healthy skin. Best of all, the scarified tissues show often, after complete cure, no signs of a scar. For the operation, Vidal recommends small lancet-shaped needles, with which several incisions 1–1½ m.m. are to be made. Deeper incisions may give rise to scars. The scarifications should be parallel, and 2 m.m. apart, and new ones should be parallel with the earlier ones as soon as these latter have healed. Small nævi may be cured in one sitting; larger, especially upon the face, need several such. The drawbacks are pain and bleeding. For the former local anæsthetizing may be applied, and even this is not always needed. For the bleeding, touchwood or German tinder may be employed; liquor ferri is rarely required. Should the patient feel the loss of blood, the intervals between the operations may be extended. When the bleeding has ceased it is well to wash the parts with a fine, moist brush, to prevent clotting in the incisions. Vidal has in several cases needed 20 sittings for a single nævus, and considers that the result more than compensated for the time and trouble.—*Jour. de Med. et Chir. Pract.—Annales de la Societe de Med. de Gand. V. Livraison*, 1878.—*Allg. Med. Centr. Z'g*, 14 *Sept.*, 1878.

*Treatment of Lupus by Scarification.*—Dr. Selongt, in his thesis upon the pathological anatomy of lupus and its treatment by linear scarification, compares the merits of this method of Vidal with those of treatment by scraping of Volkman. Dr. T. Veiel was the first to employ the former method. Dr. B. Squire has conjoined both with advantage. Vidal adopts Veiel's method with good success. It does not prevent relapses; it does arrest the course of lupus, and cause its disappearance in a comparatively short time. The skin is locally anæsthetised; then with a needle like a cataract needle linear, parallel incisions are made as close to each other as possible. Similar cross cuts are then made, leaving the skin divided into lozenges about two min. broad. These incisions must penetrate the whole thickness of the skin, a rule which it is easy to observe, as the sound and diseased tissues differ markedly in consistency. The whole surface is to be thrown off, so there need be no fear of too many scarifications; hemorrhage is inconsiderable. Iodoform is then every morning powdered upon this cut surface which cicatrizes in a week, when the process may be repeated. Every lupus nodule requires on an average six or seven such scarifications. The scar is flat, slightly depressed, and its redness gradually diminished. The dermatologist must be ready to repeat the operation the moment signs of a relapse appear. Dr. Selongt thinks that the subacute inflammation set up in the neoplasm destroys the old or possibly segmenting cells, while the embryonic ones with the connective tissue are stimulated to the formation of a cicatrix. This method is adapted to ulcerative and to erythematous lupus. Large surfaces must be treated by small islands at a time which should be at first in the periphery of the patch, thus arresting more speedily the progress of the disease. This method as well as that by the curette have each their advantages, the latter, however, would seem preferable for that variety where the formation is considerable. The cases in which each is superior, must be decided in time when the relapses and cicatrices have been more studied.—*Du Lupus, anotomic pathalogique et traitment par la methode dss scarifications lineairés, par le Dr. Selongt: These de Paris*, 1877: *ann de Dermatolojic et de syphiligraphic. T. IX. No.* 4, '77-78.

*Clinical Distinctions between Gummata of the Skin (Lupus Syphiliticus) aud Lupus Vulgaris*—Zeissl admits the slight difference, microscopically speaking, between gummata and the nodules of lupus vulgaris, but schematizes as follows the clinical difference for purposes of diagnosis:

Gummata (syphilitic) of Skin.—Localized, granulation tumors, of different sizes, causing caries and necrosis of bone and cartilage.

Location.—Cutis, subcutaneous cellular tissues.

Painful on pressure.

Commonly accompanied by ozœna syph.

Large and deep scars remain.

Is due to inherited taint. The velum palati is generally ulcerated.

Lupus Vulgaris.—Small, disseminated, reddish-brown nodules, which exfoliate. Intervals infiltrated.

Peripheral re-action, or ulceration, or erysipelatous swelling.

Nodules and ulcers nearly painless.

Bones of nose commonly unaffected.

Usually tendinous cicatrices.

Generally no ulceration of the velum.—*Reprint from the Annual Report of the Vienna Gen. Hospital for* 1877.

*Spedalskhed* (Norwegian leprosy.)—Dr. Rabe, having visited Norway, lectured upon leprosy before the Physiological and Therapeutical Association, of Dresden. He regards lepra as a disease *sui generis*, but modified by climate, race, general customs and personal habits. It is to be distinguished from radesyge, from the Dithmæsisch disease and from scarlievo, all of which are of syphilitic origin. The prognosis is absolutely unfavorable, death almost invariably occurring after prolonged illness, though deferred by good treatment, change of climate, etc. The tubercular form rarely lasts more than nine years; the anæsthetic from six to twenty-four. Death may be speedy from pyæmia. In a few cases the diseased process has arrested itself and the ulcers have cicatrized.

Of the many thousand lepers treated during the last fifteen years in the three hospitals at Bergen, only fifty-six can be called cured. The etiology is very doubtful. Norway has the greatest per cent. of lepers of all the countries in the world Still it is diminishing while the population, in spite of emigration to America, is increasing. The whole country is districted; each district has a physician who visits it, hunts up the lepers, sends them to the hospitals and a list of them to the government, which, in spite of its poverty, spends annually large sums for the relief of such unfortunates. Twice a year the general inspector makes a tour of the whole country. Lepra is an endemic disease, and confined at present, in Norway, to the west coast. Lepra is hardly acquired; nearly every case being traced to hereditary taint, and this more often from the mother than from the father. Atavism is not uncommmon. Danielssen has seen lepra transmitted to the fourth generation.

It rarely occurs before the sixth year, and usually not until after puberty. When occurring in childhood, it interferes with subsequent sexual development; it is more common in men than in women; it rarely appears primarily after the fortieth year of life; if inherited, it occurs before puberty, if acquired, later. The poison must work for years upon a healthy person before the disease becomes apparent. Some authors hold that it is the pre-disposition to lepra and not the disease itself which is inherited. Boeck found lepra in the descendants of leprous Norwegians who had emigrated to America many years before. Danielsson thinks there is some connection between lepra and tuberculosis.

How the first case originated in Norway is not known.

The disease was there before the crusaders returned from Palestine. How to relieve the present cases is an equally doubtful matter. There is no specific against lepra. Emigration gives the best results. The next best come from proper hygienic conditions; cleanliness and soothing applications, locally, are of service and incutaneous injections of morphine for pain. Every internal remedy ever suggested, has been tried at Bergen with equal lack of success. —*Arch de Heilkunde*, 15*th June*, 1878.

*Leprosy in Spain.*—A correspondent of the *France Medicale* writes from Madrid, in October, 1878, that leprosy has appeared in various districts of Alicante, calling even for the erection of special hospitals. In Valencia, during one year 116 cases of leprosy occurred; of these 71 were fatal; of the non-fatal cases 17 were women. Moreover, a majority of the cases were probably never brought to light, since those attacked endeavor in every way to hide the fact from even their nearest relations as something disgraceful. The disease goes by the title now of the "Moorish disease," now of "the disease of Saint Lazarus." In Valencia and Alicante the malady appeared under two forms: "the tubercular, or lepra græcorum," and the usual "lepra anæsthetica, or lepra hæbrorum." Recovery "very rare." There is a special hospital for lepers near Valencia, and patients refusing to go there are isolated and subjected to the strictest hygienic regulations.—*Allg. Wien. Med. Z'g, Oct.* 29, 1878

*Leprosy.*—Dr. J. Labonte, of Mauritius, gives, in an admirably terse and comprehensive paper, several cases of leprosy. The anæsthetic variety is the less repulsive, lacking the crude or suppurating tubercles and the leonine appearance of the face, characteristic of the tubercular form. This, however, is severe in its effects, the patient being almost invariably rendered helpless. When it first sets in bullæ show themselves, principally on the hands and feet, fingers and toes, where, after a short time, they burst and leave behind ulcers, which usually heal more readily than in the other form, except those which are on the plantar aspect of the foot. Accompanying this there is numbness, varying in degree and extent, and, if a close inspection be made at the time, there will be invariably found upon the extremities discolored patches of skin of a dull white or waxy yellow hue, having in some instances a scaly appearance, and in others a sort of metallic lustre. Like patches are not uncommonly met with upon the face also. Muscular atrophy, which, perhaps, is the earliest symptom in this form, is now apparent and progresses steadily, involving principally the extensor muscles of the hands. First the little and ring fingers are flexed then the remaining ones, flexion beginning at the distal phalanx and extending gradually to the second and first, the hand thus becoming unserviceable. Ulceration at the joints may cause the phalanges to fall off, reducing hands and feet to mere stumps. So great is the waste of muscular tissue that the palm of the hand is soon converted into a cavity of skin and bone, the inner and outer borders closely approximating. Muscular atrophy is not general in all cases, but is mostly confined to the extremities. The countenance, as a whole, is seldom much altered, except where, from paralysis, there is eversion of the lower eyelid, with epiphora and photophobia and dragging of the face to one side, with wrinkling of the skin, as in old age, due to muscular atrophy. Blindness may occasionally supervene. Appetite, sleep and general health may be satisfactory, even to the patients. Other special senses rarely affected. Hair and eyebrows may fall. This former occurs in both sexes, irrespective of age, and developes more slowly and with less suffering than the tubercular form, spontaneous cures being also more frequent. The two forms may be "mixed" upon the same patient, or a parent may have one form, the offspring the other. Pigmentary changes occur from possibly some impairment in the functional activity of the vaso-motor system of nerves. The disease is not only hereditary, even atavism is shown by one case, where the offspring (a girl) of one of a patient's brothers, a stout, strong man, in perfect health, married to a woman equally stout and healthy looking, took on the disease at the age of eight years. *Edinburg Med. Jour., Nov.* 1878.

*Nerve Stretching in Anæsthetic Leprosy.*—Dr. E. Lawrie, of the Bengal medical service, reports in the *Indian Medical Gazette* for September, the case of a man aged 40, who was admitted into the Medical College Hospital at Calcutta, on July 1st, with anæsthetic leprosy. On admission there was complete loss of sensation all over the patch. The patient could only grasp feebly with the right hand, and the ulnar nerve was very much thickened from below the inner condyle of the humerus to about half way up the arm. The nerve was stretched the same day under chloroform.

No regular notes of the case were taken afterwards. The patient attended very irregularly, and would never admit that his condition was improved, though the affected skin became visibly healthier. After a long interval he presented himself on August 5th, and it is recorded that "the skin of the hand and forearm is uniformly healthy; sensation is perfect throughout the area that had been anæsthetic, and the thickening of the ulnar nerve has entirely disappeared. The patient admits that the tingling and pain no longer trouble him, and that his hand is much stronger."

Dr. Lawrie has stretched the ulnar nerve in about thirty cases of anæsthetic leprosy. In every case the operation was followed by benefit, as far as the area supplied by this particular nerve was concerned, which appeared likely to be permanent. The patients ceased attending the dispensary whenever the relief they experienced seemed to them decisive, and therefore no notes of their final condition were obtainable, except in the present instance.—*London Med. Record*, *Nov.* 15, 1878.

*Anæsthetic Leprosy.*—Tilbury Fox reports the case of a boy of 17, of English descent, born in Bombay, no hereditary taint. Disease began at the age of 11 as brown marks in patches on body, limbs, face, hands and feet, without subjective symptons; previous health good. Treated by Dr. Bhan Daji with some essential oil externally and internally. After several months he became ill, his strength left him, his hands, feet and face swelled and sores broke out upon his legs. He gradually recovered and, after two years, seemed quite well except some disordered sensation and numbness about the elbow and right ear. He then came to England where he has always lived well, and the lepra for about three years and a half remained in a quiescent state. At present he suffers from an acute recurrence of the disease (leprosis), beginning two or three months ago as a brownish discoloration about the cheeks and nose. These spots are now generally diffused on the skin and are not mere maculations as in the former attack, but exhibit distinct structural changes in the skin texture. Other symptoms, as at first, of eruption with slight deposit in skin of nerve lesion, knotty state of and anæsthesia of superficial nerve, marks in circular blotches, dry, scaly and withered, with dirty white or faint red centres and more conspicuous, reddish-yellow, well defined, semi-psoriatic looking edges; bullæ also on the feet. The progress of these cases is always slower than that of the tubercular form. There is no specific for lepra. The disease may be ameliorated by the use of cashew, gurjam or chaulmugra oils. Patients should leave climates where lepra is endemic for temperate and bracing ones; hygiene is of the greatest importance. Fox has faith in the value of quinine pushed to extreme doses, mineral acids and chlorate of potash with gurjam infricted twice daily.—*Medical Times and Gazette, Dec.* 21, 1878.

*Chaulmugra oil in the treatment of Leprosy.*—Dr. David Young reports six illustrative cases out of between fifty and sixty treated for leprosy at the Mission Hospital, Bombay, during eighteen months. The patients were all adults, the proportion of males to females about three to one. The treatment was by chaulmugra oil, alone or combined with the tincture internally, or a liniment externally of psoralea corylifolia, a leguminous plant abounding in the Konkan and Deccan. The forms of leprosy noted were, macula, 4; anæsthetic, 23; tubercular, 15; mixed, 11. Out of the six cases reported, but one obtained no apparent benefit. The other five, after a treatment of from six months to a year, were all improved in health with an increase in weight and more or less new growth of hair. Dr. Young sums up as follows:

1. In the macular and in the early stage of the anæsthetic forms of leprosy, the chaulmugra appears to be of decided value.

The oil should be given at the outset.—*The Practitioner, Nov.*, 1878.

*Multiple Melanotic* (?–Rep.) *Sarcoma of Skin.*—In the hospital reports of the Toronto General Hospital, occurs a very brief account of a cure of multiple sarcoma. A man of 64, reporting a cousin with cancer of the breast, noticed thirty years ago (say in 1847) a black, pin-head sized papule an inch to the left of the navel. In the summer of 1876 it became as large as a currant. In the fall of 1876 it became chafed, "discharged blood and burst through the skin," and increased in size, having been cauterized. In the spring of 1877 it was strangulated with horse hair, but grew again, uniting with three adjoining tubercles of a similar character, which also appeared at this time, to form one large tumor, which measured November 30, 1877, 10x5x3.75 c. m., was soft, fungoid and though non-discharging, of a most offensive odor, causing even gastric disturbance. Slight lancinating pains at times. Early in October, 1877, some two hundred movable or immovable, hard, colorless, tubercles from pea to cherry size, formed under the integument all over the body; "those situated near veins of a pinkish color, and some of these have burst through the skin." General health otherwise good. The large tumor was removed by Dr. Aikens, by means of the galvanic cautery, and the patient has since been progressing favorably without the aid of medicines. Portions of the tumor under the microscope showed the characteristic round and spindle cell formation of sarcoma with in (parts) some pigmentation.—*Canadian Journal of Medical Science, vol. III, No.* 2, *p.* 58.

*Rodent Ulcer.*—Thin has examined microscopically a cancerous ulcer of forty-three years duration, combining the chief features characteristic of rodent ulcer, which was removed by excision by Sir James Paget, at which time it covered the whole scapula; and also a rodent ulcer measuring 3x4 inches, removed with the knife by Mr. Morrant Baker. Under the border of the ulcer, in both cases, and, for a very small difference, under the epidermis of the margin, clusters of cells separated the bundles of connective tissue, and grew most luxuriantly toward the surface. The clusters were largest in the upper strata of cutis, and caused the progressive ulceration by obliterating the connective tissue and blood vessels of the papillary layer. The centre was composed, in each case, of a substratum of unaltered connective tissue of the cutis covered by an amorphorus substance, containing blood vessels and large numbers of colorless blood cells. Sparsely scattered through this tissue, were patches of the above mentioned cell-clusters. In the sections examined, the only changes found in the rete mucosum, sebaceous glands, and hairs were retrogressive, thus contrasting with what is found in epithelioma. Diseased sweat glands were not found. The results of these examinations show that in some, at least, of the cases distinguished by the name of rodent ulcer, we have to do with a special pathological condition having no near relation to epithelioma. Paget's case resembles the one described by Verneuil, in which the departure of morbid action was definitely traced to the sweat glands. This, and the evidences of a tendency to new cell formation in the sweat glands observed in the former one, points to the strong probability that the rodent ulcer of English surgeons and the adenoma of the sweat glands of the French surgeon, are one and the same disease.—*Reprint from the transactions of the Pathological Society of London for* 1878.

Differential diagnosis of multiple carcinoma and of sarcoma melanodes from gumma syphiliticum, Zeissl summarizes as follows, the distinctions between gummata and carcinomatous formations:

*Gumma Syphiliticum.*

Spherical nodules.

Most common on face and extremities.

Nodules frequently re-absorbed.

Ulceration frequent.

Microscope shows a scanty mucilaginous intercellular fluid. The tissue approximates to that of the embryonic state.

*Multiple carcinoma; sarcoma melanodes.*

Flattened formation.

Most common on trunk, rare on face.

Re-absorption very rare.

Skin generally remains intact.

In multiple carcinoma a white, black or speckled medullay mass; in sarcoma melanodes elementary fibrous tissue loaded with pigment.—*Reprint from Annual Report of the Vienna General Hospital, for* 1877.

